# Synergistic effect of nanofat and mouse nerve‐growth factor for promotion of sensory recovery in anterolateral thigh free flaps

**DOI:** 10.1002/sctm.20-0226

**Published:** 2020-10-11

**Authors:** Shune Xiao, Fengling Zhang, Yongjian Zheng, Zhiyuan Liu, Dali Wang, Zairong Wei, Chengliang Deng

**Affiliations:** ^1^ Department of Plastic Surgery Affiliated Hospital of Zunyi Medical University Zunyi People's Republic of China

**Keywords:** anterolateral thigh flap, nanofat, nerve‐growth factor, sensory recovery

## Abstract

Anterolateral thigh (ALT) free flaps are widely used for reconstruction, but poor sensory recovery of the flap tissue can cause unsatisfactory outcomes and poor function. Adipose‐derived mesenchymal stem cells (ADSCs) promote neural regeneration, but the clinical use of stem‐cell therapy has been limited by lack of regulatory approval. Nanofat is an autologous product that is prepared mechanically from harvested fat. It is enriched in ADSCs and does not contain any exogenous substances. The developmental and adult neurobiology of nerve‐growth factor (NGF) are well investigated, and mouse (m)NGF has been used to promote recovery following peripheral nerve injury. We investigated the promotion of nanofat and mNGF as either mono‐ or combined therapy on the sensory recovery of ALT free flaps. We found that nanofat and mNGF had a synergistic effect on sensory recovery that was associated with stimulation of angiogenesis and neurogenesis. Nanofat combined with mNGF was better at promoting neural regeneration and improving sensory recovery than treatment with either agent alone. The results provide a theoretical rationale for further study of the clinical use of nanofat combined with mNGF to promote the sensory recovery of ALT free flaps.

## Graphical abstract

Schematic of the functional mechanism of nanofat combined with mouse nerve growth factor (mNGF). Nanofat and mNGEF as either mono‐ or combined therapy on the sensory recovery of anterolateral thigh free flaps was investigated. Nanofat and mNGF had a synergistic effect on sensory recovery that was associated with stimulation of angiogenesis and neurogenesis.

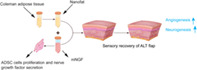

## 1 INTRODUCTION

Song et al described the vascular anatomy and surgical applications of anterolateral thigh (ALT) free‐flap procedures in 1984.^1^ The desirable characteristics of the ALT free flap include its large skin surface area, suitable pedicle caliber, long vascular pedicle, and possible harvesting as a chimeric flap.^2^, ^3^, ^4^ Successful surgical repair and flap survival after transplantation are primary concerns, but reducing complications at the recipient site and achieving perfect reconstruction have long been the ultimate goal of reconstructive surgeons. Sensory disorders associated with ALT free‐flap reconstruction can result in poor functioning of repairs in regions such as the face, hands, feet, and perineum.^5^, ^6^, ^7^ Achieving a perfect repair with accelerated sensory recovery of recipient site has become a challenge to reconstructive surgeons.

Free perforating flap transplantation involves both denervation and reinnervation. In addition to nerve anastomosis during surgery, nerve fibers regenerate in the free flaps mainly from the surrounding area and base, but the presence of few neurofilament‐positive sensory nerve fibers in the dermis or epidermis may result in poor sensory recovery. The harvesting of ALT free flaps with donor nerves and subsequent microsurgical anastomosis with the recipient nerves promotes sensory recovery.^8^, ^9^ However, numbness and sensory disorders may occur because of the denervation of the donor site, and neuromas that occur at sites of nerve anastomosis may cause pain. Therefore, alternative safe and effective approaches are required. Previous studies have demonstrated that Schwann cell (SC) transplantation can accelerate neural regeneration and promote recovery of neural function but that implementation is difficult.^10^ Sacrifice of functional nerves during SC isolation, problems associated with culture and expansion of SC populations in vitro, immunologic rejection, and proliferation and survival of SCs after transplantation into the injury site all decrease the feasibility of transplantation.^11^, ^12^ Recent studies have found that mesenchymal stem cells (MSCs) can promote neural regeneration and may be an ideal alternative.^13^ Adipose‐derived mesenchymal stem cells (ADSCs) have advantages compared with other kinds of MSCs that make them ideal seed cells for tissue regeneration and repair. ADSCs are easily harvested and are abundant in human adipose tissue. They continuously renew and are less immunogenic than other MSCs because they lack MHC class II or costimulatory molecules.^14^, ^15^ In animal studies and clinical trials, secretion of multiple nerve‐growth factors following local transplantation of ADSCs promoted peripheral nerve repair and differentiation of neuronal cells.^16^, ^17^, ^18^, ^19^, ^20^, ^21^, ^22^ The novelty of stem cell therapy and the need for regulatory approval have limited the available clinical applications, but Tonnard et al have described the preparation of nanofat, which is enriched in ADSCs, obtained by a purely mechanical procedure, and can be administered by injection with a 27 gauge needle.^23^ Nanofat is an autologous product that does not contain any exogenous substances. Previous studies have described the successful use of nanofat for tissue repair and regeneration, anti‐aging treatment, and the treatment of scars.^24^, ^25^ There are currently no reports of the application of nanofat for nerve regeneration after free perforating flap procedures.

Nerve growth factor (NGF) regulates the survival, growth, and differentiation of nerve cells in the peripheral and central nervous systems both during development and afterward.^26^ In China, mouse (m)NGF has been used clinically to treat peripheral nerve injury,^27^, ^28^, ^29^, ^30^ but there have been no reports of its ability to promote nerve regeneration after free perforator flap procedures. This study investigated the use of nanofat and mNGF alone or in combination to promote sensory recovery after free‐flap surgery. The in vitro effect of mNGF on the proliferation of ADSCs was determined by a Cell Counting Kit‐8 (CCK8) assay. Nerve growth factors present in the culture media of mNGF‐treated ADSCs and potentially responsible for this stimulatory effect were assayed.

**Lessons learned**

• Nanofat plus mouse nerve growth factor (mNGF) injected into the deep dermis of anterolateral thigh (ALT) free flaps promoted sensory recovery.
• The mechanism may be associated with stimulation of angiogenesis and neurogenesis.
• Injection should be conducted when the flap has established a good‐working microcirculation.
• The optimal mNGF solution/nanofat ratio was 1:9 and the optimal injection volume was 0.5 mL/cm^2^.

**Significance statement**

Nanofat and mouse nerve growth factor (mNGF) had a synergistic effect on sensory recovery of anterolateral thigh (ALT) free flaps that was associated with stimulation of angiogenesis and neurogenesis. The results provide a theoretical rationale for further study of the clinical use of nanofat combined with mNGF to promote the sensory recovery of ALT free flaps.

## 2 MATERIALS AND METHODS

### 2.1 Patients and ethical approval

Sixteen patients, 10 men and 6 women, were enrolled at the Affiliated Hospital of Zunyi Medical University between June 2017 and June 2018, 4 months after ALT free‐flap transplantation. The average age of the patients was 28.6 years (range 18‐35). The average BMI of the patients was 21.6 kg/m^2^ (range 18.5‐23.9). Five had upper‐extremity and 11 had lower‐extremity ALT flap transplantations. The flap areas ranged from 90 to 150 cm^2^, all were performed without nerve anastomosis, and no flaps were surrounded by obvious hypertrophic scars. None of the patients had underlying vascular disease, hypertension, or diabetes. The flaps were divided into two halves along the midline, for a total of 32 experimental samples, and the patients were randomly assigned to treatment with nanofat combined with mNGF (n = 8), nanofat alone (n = 8), mNGF alone (n = 8), or saline alone (n = 8). The study was registered at the Chinese Clinical Trial Registry (ChiCTR2000031057, http://www.chictr.org.cn/searchprojen.aspx). All participants provided written informed consent before enrolling in the study. The protocol was approved by the Ethics Committee of the Affiliated Hospital of Zunyi Medical University.

### 2.2 Preparation of nanofat

Nanofat was prepared by the Coleman technique as previously described and with the patient under anesthesia.^23^ Briefly, adipose tissue was harvested from the lower abdomen after infiltration with 1000 mL of Ringer's solution containing 15 mL of 5% xylocaine, and adrenaline 1/100 000. The harvested tissue was filtered, cleaned, washed with Ringer's solution, depleted of thick fibrous tissue, and emulsified by 30 transfers between two 10 mL syringes connected by a Luer‐Lock fitting with an internal diameter of 2.4 mm. After emulsification, the fatty liquid was filtered through a sterile nylon cloth and collected in a sterile container.

### 2.3 Treatment

**FIGURE 1:**
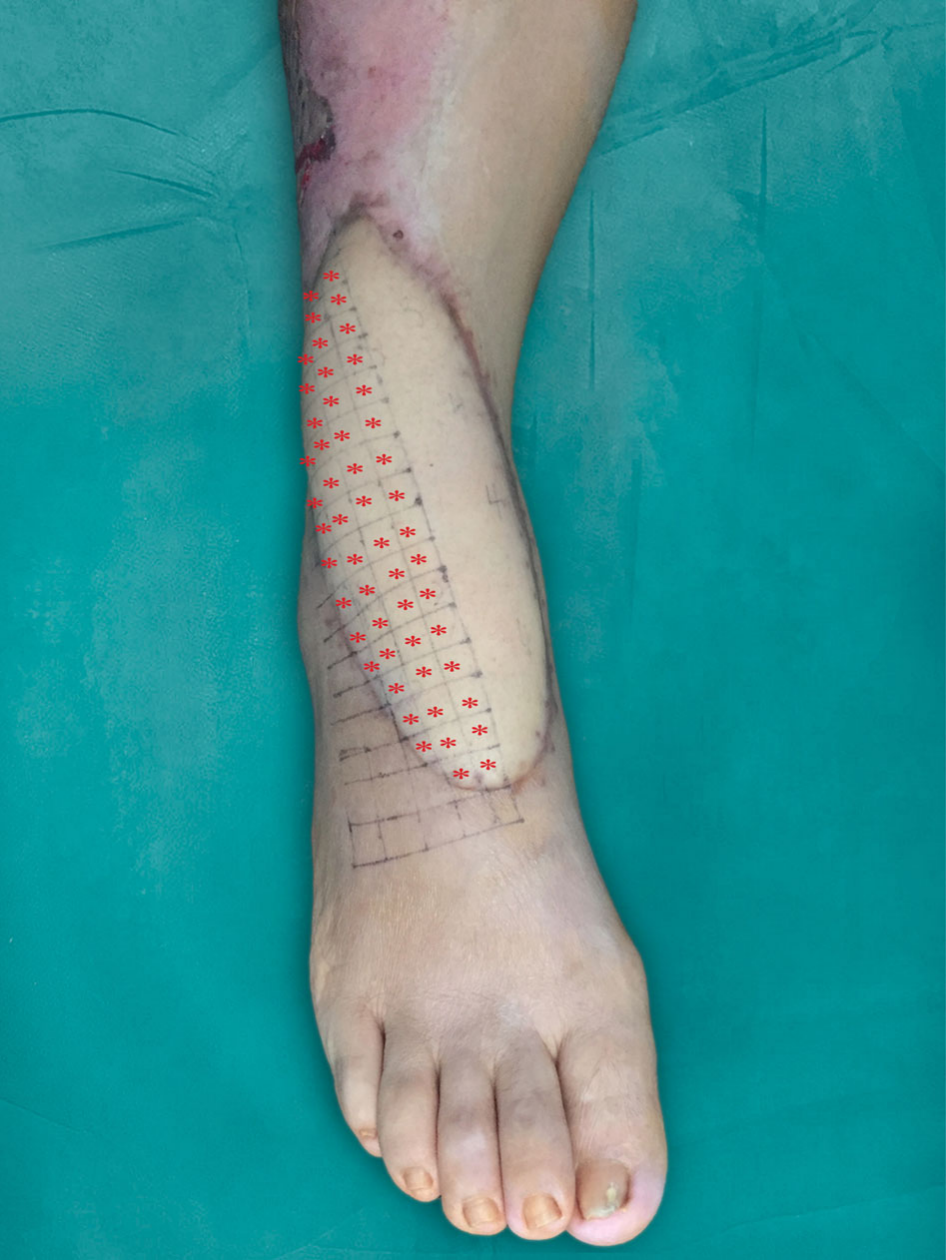
Indicated injection areas on surface of a flap. The flaps were marked with 1 cm squares and injection areas were marked by using red symbols. The flaps were given a slow injection of 0.5 mL/cm^2^ suspension or saline into the deep dermis

A 1:9 (vol/vol) mixture of 18 μg mNGF powder (Shandong Weiming Biomedical Co., Ltd. Shangdong, China) in 2 mL of sterile saline and 18 mL nanofat was prepared and used to treat patients in the mNGF + nanofat group. A 1:9 (vol/vol) mixture of mNGF solution and sterile saline was prepared and used in mNGF group. Equal volumes of nanofat or sterile saline were given to nanofat only and control patients. The flaps were marked with 1 cm squares as shown in Figure 1 and was given a slow injection of 0.5 mL/cm^2^ suspension or saline into the deep dermis with a 27 gauge needle. The blood flow of the flap was observed, the color and capillary filling test was used to judge the blood supply of the flap. The color of the flap‐were ruddy for normal blood supply, and pale for ischemia performance. The local capillary filling time of the flap was 1 to 2 seconds for normal blood supply, and more than 3 seconds indicates insufficient blood supply to the flap. Once the flap blood supply disorder occurs, the injection should be stopped immediately and emergency measures such as the use of vasodilator drugs and local physical therapy should be adopted.

### 2.4 Sensory evaluation before and after treatment

Before, 2, and 10 months after treatment, sensory function was evaluated by static two‐point discrimination (2PD) and Semmes‐Weinstein monofilament (SWM) tests. The test point was at the center of each experimental sample. SWM test was performed by using a set of monofilaments which have varying thicknesses (North Coast Medical, Inc., California). 2PD test was performed by using a *DellonDisk‐*Criminator tool (North Coast Medical, Inc.). Between‐group differences were evaluated by Chi‐square tests. *P* values <.05 were considered statistically significant.

### 2.5 Histologic analysis

Before and 2 months after treatment, full‐thickness skin samples at the center of each experimental sample were obtained from the flaps using a 5 mm circular punch. Vascularization and neuroregeneration were assayed by immunohistochemical staining of CD31 and S100. The skin samples were fixed in 4% paraformaldehyde (Sigma‐Aldrich, St. Louis, Missouri) for at least 24 hours, embedded in paraffin, and sectioned at 4 μm. Deparaffinized sections were blocked with 1% bovine serum albumen (Sigma), and incubated with anti‐CD31 (1:250; Abcam, Cambridge, UK) or anti‐S100 (1:250; Abcam) primary antibodies overnight at 4°C. The sections were then incubated with secondary antibody (1:250, Abcam) for 30 minutes, stained with diaminobenzidine (Invitrogen, Grand Island, New York) and hematoxylin, dehydrated, cleared, and mounted. Percentage‐positive expression of S100 and CD31 was determined in five high‐powered fields per sample by ImageJ software (https://imagej.nih.gov/ij/).

### 2.6 Isolation, culture, and flow cytometry assay of ADSCs

Human abdominal subcutaneous adipose tissues were harvested during liposuction procedures after obtaining signed informed consent in the Department of Plastic Surgery, Affiliated Hospital of Zunyi Medical University, China. ADSC isolation and culture was performed as previously described.^15^ Briefly, human adipose tissue was minced and digested with 0.075% collagenase type I (Sigma) for 45 minutes at 37°C. After digestion, an equal volume of Dulbecco's modified Eagle's medium (DMEM; Gibco, Carlsbad, California) supplemented with 10% fetal bovine serum (FBS; Gibco) was added and the suspension was passed through a 200 μm mesh filter followed by centrifugation at 800*g* for 5 minutes). The stromal‐vascular fraction cell pellets were resuspended and cultured at 37°C in 5% CO_2_ in DMEM supplemented with 10% FBS and 1% penicillin‐streptomycin (Gibco). ADSCs were subcultured at 80% confluence, and passage‐3 cells were used in the study procedures. ADSCs surface marker expression was assayed by flow cytometry. Suspensions of 1 × 10^6^ ADSCs were incubated with anti‐CD90, anti‐CD73, anti‐ CD105, anti‐CD34, anti‐CD11b, anti‐CD19, anti‐CD45, or anti‐HLA‐DR antibodies (1 mg/mL; Abcam) at room temperature for 30 minutes, washed with phosphate‐buffered saline (PBS), and analyzed with a MoFlo XDP flow cytometer (Beckman Coulter, Brea, California) and Kaluza software (Beckman Coulter).

### 2.7 Multiline differentiation of ADSCs

In vitro differentiation was performed as previously described.^28^ Adipogenesis and osteogenesis were assayed on day 21 by 1% oil red O and alizarin red S staining. Chondrogenesis was assayed on day 28 by 1% alizarin blue staining.

### 2.8 ADSC proliferation assay

The effect of mNGF on ADSCs proliferation was assayed in passage‐3 cells seeded at a density of 4 × 10^3^ cells/well in 96‐well culture plates. The cells were cultured in DMEM supplemented with 10% FBS and 1% penicillin‐streptomycin containing with 0.9, 0.09, or 0.009 μg/mL mNGF for 24 or 48 hours. The proliferation of cultured ADSCs was assayed with a Cell Counting Kit‐8 (Sigma). The absorbance of culture media was measured at 450 nm using a multilabel counter (n = 3).

### 2.9 Enzyme‐linked immunosorbent assay (ELISA)

ADSCs were cultured in medium containing with 0.9, 0.09, or 0.009 μg/mL mNGF at 37°C in 5% CO_2._ After 24 hours, the medium was replaced with serum‐free medium and without mNGF. After a further 24 hours, the supernatants were collected and passed through a 40 μm syringe filter to remove cell and tissue debris. Nerve growth factor (NGF) and neurotrophin‐3 (NT‐3) in the supernatant were assayed using a Quantikine ELISA kit (Sigma‐Aldrich) following the manufacturer's instructions.

## 3 RESULTS

### 3.1 Recovery of sensory function

**FIGURE 2:**
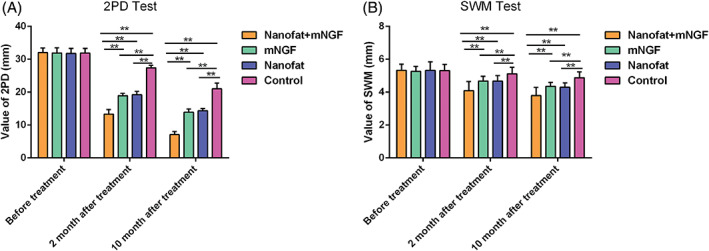
Sensory evaluation using a static two‐point discrimination (2PD) test and the Semmes‐Weinstein monofilament (SWM) test. Patients with nanofat plus mNGF had the smallest 2PD distance (A) and SWM (B) compared with nanofat or mNGF alone or control patients 2 and 10 months after treatment, which show that recovery of sensory function was better in patients treated with nanofat plus mNGF than in the other three groups. Differences in the results obtained with nanofat and with mNGF alone were not significant. **P < .05

All flaps survived well without any complications and were followed‐up for 10 months after surgery. As shown in Figure 2, recovery of sensory function was better in patients treated with nanofat plus mNGF than in the other three groups. There were no significant differences in the static 2PD or SWM test results in each group before treatment (*P* > .05). At both 2 and 10 months after treatment all three treatment groups had better 2PD and SWM test scores than the control patients (*P <* .05). Patients treated with nanofat plus mNGF had an average 2PD of 13.3 mm (range 11.4‐15.6 mm) and an average SWM test score of 4.09 (range 3.61‐4.93) at 2 months after surgery and an average 2PD of 7.1 mm (range 6.0‐8.8 mm) and SWM test score of 3.79 (range 3.22‐4.56) at 10 months.

### 3.2 Nanofat plus mNGF increased neoangiogenesis in flaps

**FIGURE 3:**
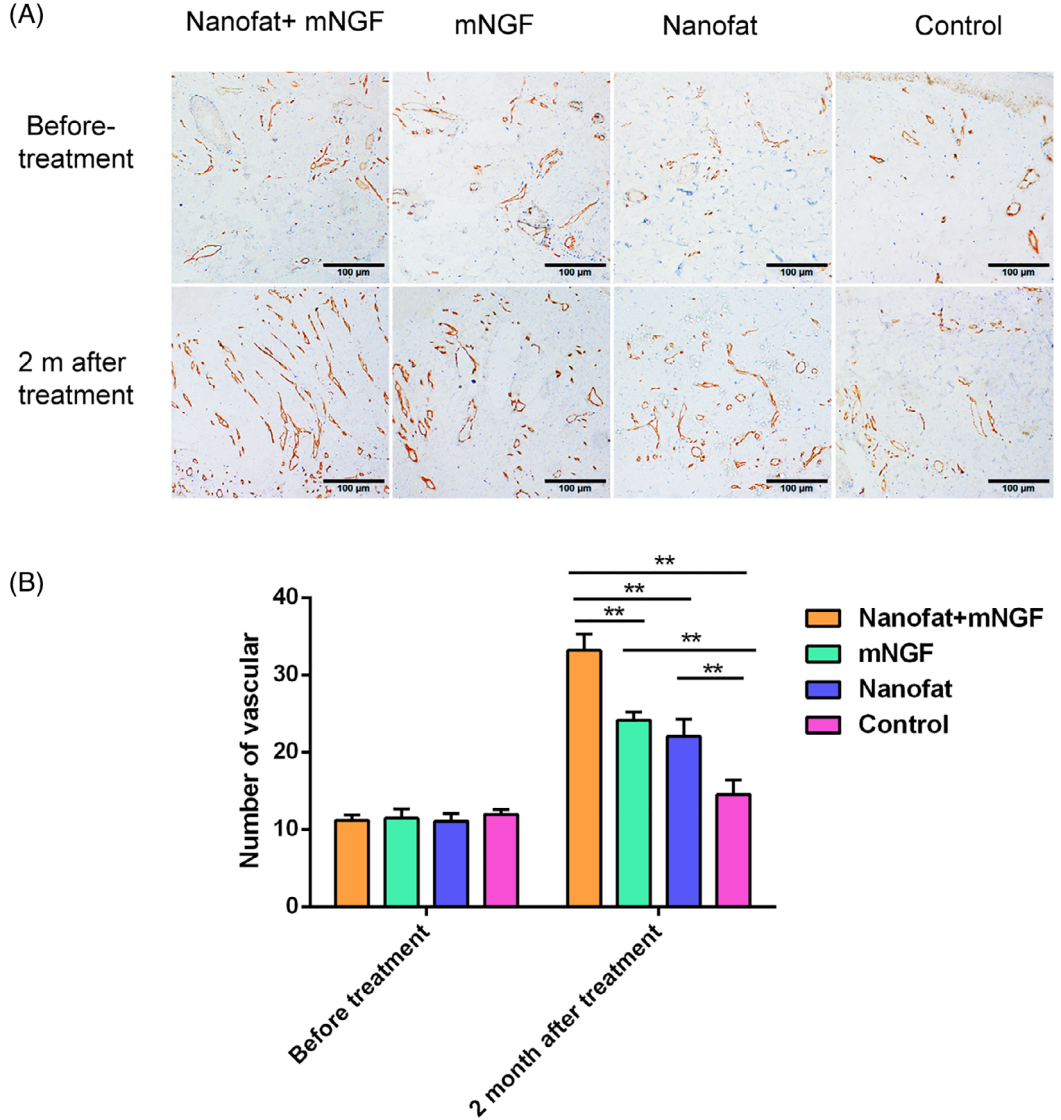
Angiogenesis in flaps was evaluated by immunohistochemical staining of CD31 at 2 months after treatment. There were no significant differences in the number of blood vessels in the four groups before treatment. More new blood vessels were seen in the flaps of patients treated with nanofat plus mNGF, nanofat alone, and mNGF alone than in controls. Angiogenesis was the most evident in flaps treated with nanofat plus mNGF. Differences in angiogenesis seen in flaps treated with nanofat and mNGF alone were not significantly different. **P < .05

Angiogenesis was evaluated in flaps by immunohistochemical assay of CD31 expression in blood vessel endothelial cells at 2 months after surgery (Figure 3). There were no significant differences in the number of blood vessels in the four study groups before surgery. More blood vessels were seen in flaps treated with nanofat and/or mNGF than in those treated with sterile saline. Flaps treated with nanofat plus mNGF had more new blood vessels than those treated with nanofat or mNGF alone.

### 3.3 Nanofat plus mNGF increased neurogenesis in flaps

**FIGURE 4:**
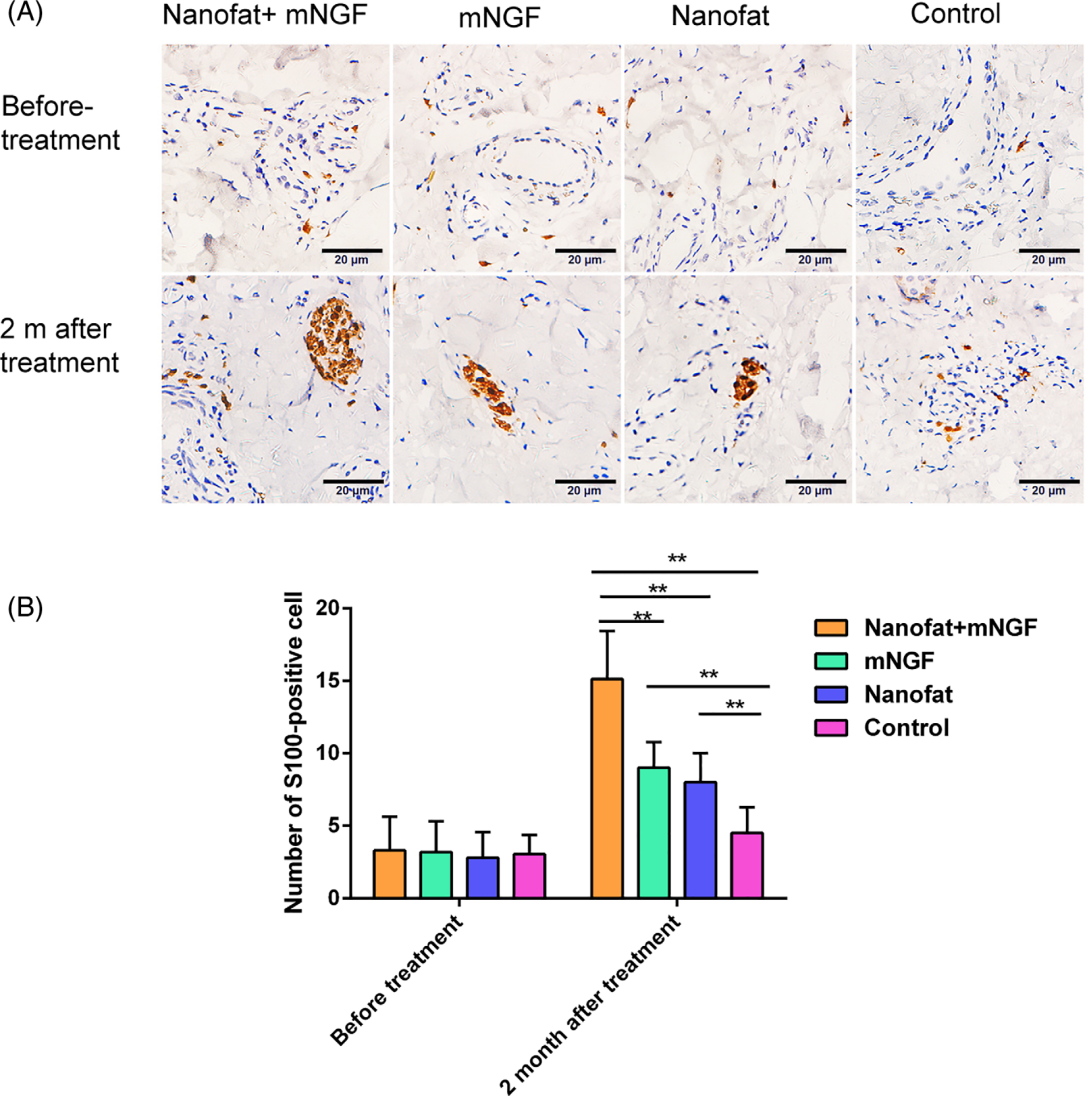
Neuroregenesis in flaps was evaluated by immunohistochemical staining of S100 at 2 months after treatment. Few S100^+^ cells were seen in any flaps before treatment. More Schwann cells were seen in flaps treated with nanofat plus mNGF, nanofat alone, and mNGF alone than in controls. More Schwann cells were seen in flaps treated with nanofat plus mNGF than in those treated with nanofat alone and mNGF alone. **P < .05

Neurogenesis in flaps was evaluated by immunohistochemical staining of S100 in Schwann cells at 2 months after surgery (Figure 4). Few S100^+^ cells were seen in the four study groups before treatment, but more Schwann cells were seen in flaps treated with nanofat and/or mNGF than in controls and more were seen in flaps treated with nanofat plus mNGF than in those treated with nanofat or mNGF alone.

### 3.4 mNGF promoted ADSC proliferation and nerve growth‐factor secretion

**FIGURE 5:**
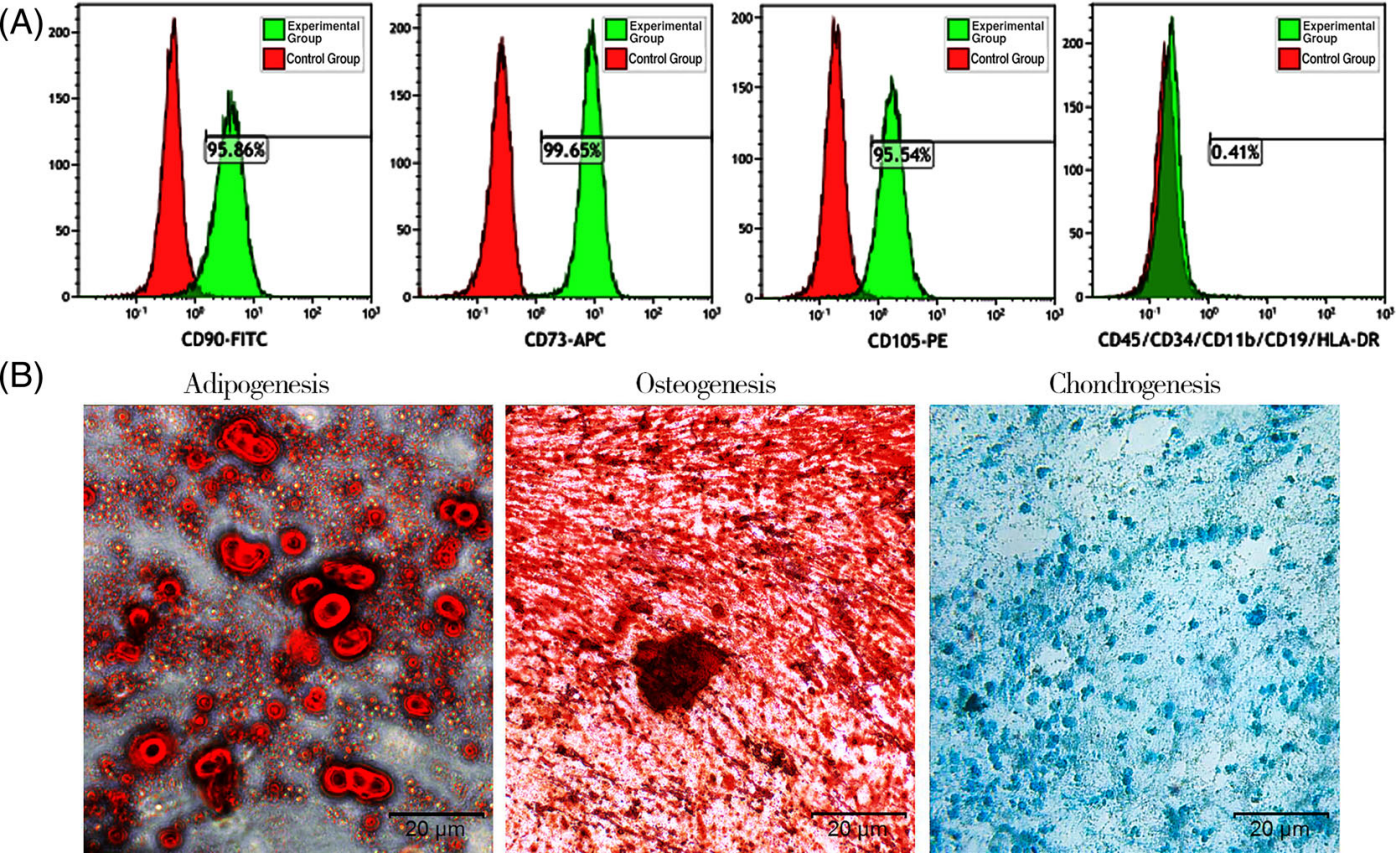
Characterization of adipose‐derived mesenchymal stem cells (ADSCs). A, Flow‐activated cell sorting analysis of ADSC markers. ADSCs were >90% positive for CD90, CD73, and CD105, and negative for CD45, CD11b, CD19, CD34, and HLA‐DR. B, Differentiation of cultured ADSCs into osteocytes, adipocytes, and chondrocytes. Cells were stained for calcium by alizarin red, lipid droplets by oil red O, and acid mucopolysaccharide with alizarin blue

**FIGURE 6:**
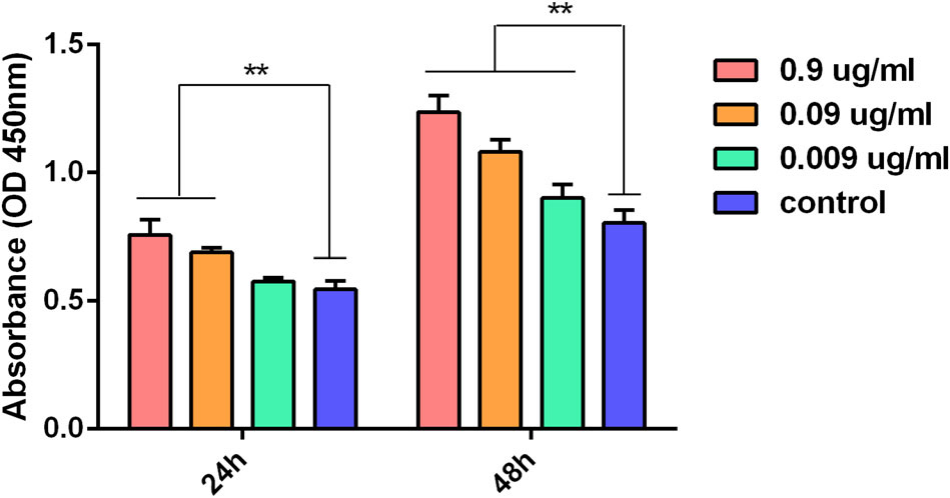
The effects of mNGF on adipose‐derived mesenchymal stem cells proliferation. mNGF increased cell viability in a dose‐dependent manner. **P < .05

**FIGURE 7:**
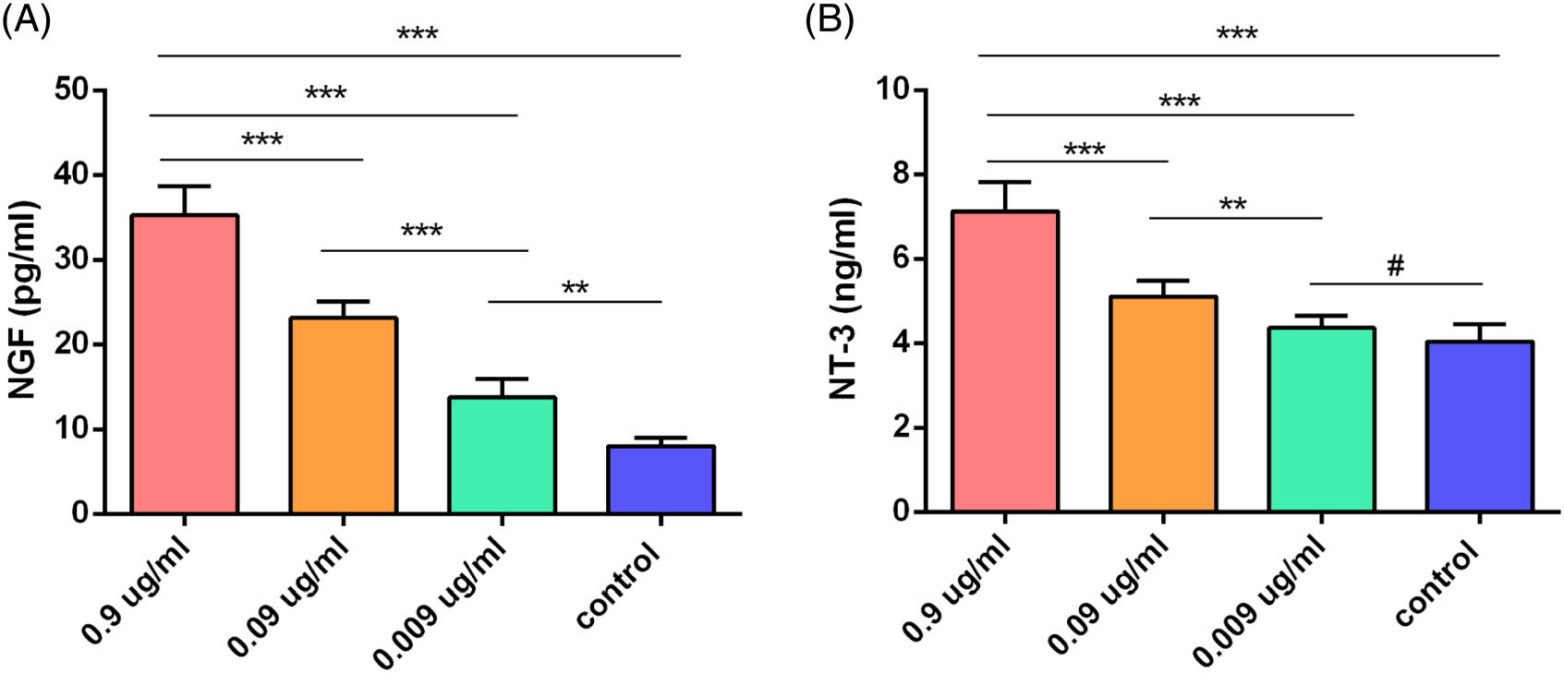
Enzyme‐linked immunosorbent assay (ELISA) of nerve‐growth factor (NGF) and NT‐3 expression induced in adipose‐derived mesenchymal stem cells (ADSCs) by increasing concentrations of mNGF. A, NGF expression was significantly higher in mNGF‐treated ADSCs than in controls and increased in a dose‐dependent manner. B, NT‐3 expression was significantly higher with 0.9 and 0.09 μg/mL mNGF compared with control cells. **P < .05, ***P < .01, ^#^ *P* > .05

The effects of mNGF on ADSC proliferation were assayed by flow cytometry and multilineage differentiation. ADSCs were >90% positive for CD105, CD73, and CD90 and negative for CD34, CD11b, CD19, CD45, and HLA‐DR (Figure 5A). Positive Oil red O, alizarin red S and alizarin blue staining confirmed that ADSCs had differentiated into osteocytes, adipocytes, and chondrocytes (Figure 5B). The dose‐dependent effects of mNGF on ADSC proliferation are shown in Figure 6. mNGF stimulated paracrine secretion of NGF by ADSCs responsible for neurogenesis in flaps. NGF levels were significantly higher in mNGF‐treated ADSCs than in controls and increased with the mNGF dose (Figure 7A). Secretion of NT‐3 by ADSCs also increased with increasing mNGF concentration. NT‐3 levels were significantly increased by mNGF at both 0.9 and 0.09 μg/mL compared with controls (Figure 7B).

## 4 DISCUSSION

Sensory recovery of free flaps is a challenge for reconstructive surgeons. In this study, nanofat plus mNGF injected into the deep dermis of ALT free flaps promoted sensory recovery that may have been associated with increased angiogenesis and neuroregeneration. Our results showed that static 2PD and SWM evaluation improved significantly in the nanofat plus mNGF group, which indicated that combination of mNGF and nanofat had a positive effect on sensory recovery. Histologic analysis of our study showed the number of Schwann cells and number of blood vessels in flaps increased in the nanofat plus mNGF group at 2 months after surgery.

This indicates that the combination of mNGF and nanofat can promote the regeneration of blood vessels and nerve fibers in flaps and accelerate the sensory recovery of flaps. Previous studies have revealed interaction relationship between blood vessels and nerve fibers in their development and regeneration.^31^, ^32^

Several growth factors have been identified as important mediators that guide angiogenesis and regulate axon terminal arborization. Among them, NGF has been proved not only to promote peripheral nerve development and regeneration, and also to promote angiogenesis.^33^, ^34^ Moreover, mesenchymal stem cells in accompany with NGF have been proved to have a synergistic effect ‐on promoting peripheral nerve repair.^35^ In vitro, our studies show that mNGF has positive effects on the proliferation of ADSCs and secretion more neurotrophic factors, which suggested a potential therapeutic applicatio with the synergism of the two.

Nanofat is a liquid fat emulsion prepared by mechanical processing and was first described by Tonnard et al in 2013.^23^ Adipocytes do not survive nanofat preparation, but nanofat does contain many ADSCs, which may be responsible for neovascularization in the recipient area following intradermal injection, and the promotion of angiogenesis that is important for peripheral nerve growth.^23^, ^26^, ^36^ As a stem cell therapy, the advantages of nanofat include simple, rapid preparation with no need of isolation and cultivation in the laboratory. Proliferation, differentiation potential, and stem cell properties are preserved in cell culture.^23^ Compared with ADSCs, nanofat is easier to prepare, more economical, and safer in clinical applications. NGF has positive effects on neuronal survival, angiogenesis, Schwann cell viability, and proliferation during the development and regeneration of peripheral nerves.^37^ mNGF, which can be extracted from the submandibular glands of male mice, and human NGF are highly homologous.^26^ Its ability to promote nerve regeneration led to approval for clinical use in China in 2003 as a treatment of peripheral nerve injury.

In this study, we chose the treatment at 4 months after ALT flap transfer, at which time the flap has established a good‐working microcirculation, and proper treatment will not cause partial or complete necrosis of the flap. Studies have present evidence that obese‐derived ADSCs show impaired migration, differentiation and angiogenesis properties, especially for those with BMI > 30 kg/m.^38^ In the study, the patients' BMI were between 18.5 and 23.9 kg/m^2^, which were in the normal range. Therefore, we think it would not affect the efficacy of nanofat. The standard injection protocol used in this study, the mNGF and nanofat volume ratio and the injection volume/cm^2^, was determined in a preliminary study. To determine the optimal injection volume, 0.3, 0.5, and 0.8 mL/cm^2^ nanofat were evaluated. At 0.8 mL/cm^2^, the flap became pale because of reduced blood flow. At 0.3 and 0.5 mL/cm^2^ the flap color remained red and without signs of an insufficient blood supply. An injection volume of 0.5 mL/cm^2^ was chosen because sensory recovery at 2 months was better than that seen following injection of 0.3 mL/cm^2^. To determine the optimal mNGF solution/nanofat ratio, both 1:4 (vol/vol) and 1:9 (vol/vol) were evaluated. Following injection of a ratio of 1:4, patients complained at the 2‐month follow‐up that the flap was hard to the touch. A ratio of 1:9 was selected because it led to a soft flap and greater patient satisfaction.

This is the first report of the use of mNGF plus nanofat to promote the sensory recovery of the free flaps. Clinical assessment was by 2PD and SWM tests. Histological assessment of recovery was by immunohistochemical staining of CD31 and S100 before and after the free‐lap procedure. Although the study was limited by a small number of cases and short follow‐up, the results did demonstrate the benefits of combined treatment with nanofat and mNGF. Evaluation of additional patients with longer follow‐up is ongoing. Moreover, more data is needed before injection of mNGF and nanofat becomes universally performed.

## 5 CONCLUSION

In this preliminary study, we investigated the sensory recovery‐promoting effect of mNGF combined with nanofat. The synergistic effect of mNGF and nanofat on sensory recovery in these 16 patients with ALT free flaps can be explained by stimulation of angiogenesis and neurogenesis. It is a very small sample size of varying subjects and flap locations and more data is needed before injection of mNGF and nanofat becomes universally examined. Although further investigation is necessary, the technique has great potential for clinical use to promote sensory recovery of free flaps.

## CONFLICT OF INTEREST

The authors declared no potential conflicts of interest.

## AUTHOR CONTRIBUTIONS

C.D.: conception and design, financial support, final approval of manuscript; S.X.: provision of study material or patients, data analysis and interpretation, manuscript writing; Z.W.: conception and design, administrative support; D.W.: administrative support; F.Z.: collection and/or assembly of data, data analysis and interpretation; Y.Z.: collection and/or assembly of data; Z.L.: data analysis and interpretation.

## Data Availability

The data used to support the findings of this study are included within the article.
